# Retraction: Shaoyao-Gancao Decoction alleviated hyperandrogenism in a letrozole-induced rat model of polycystic ovary syndrome by inhibition of NF-κB activation

**DOI:** 10.1042/BSR20181877_RET

**Published:** 2026-06-03

**Authors:** Jun-jin Liu, Rui-gang Hou, Yun-yun Shao, Zhuang-peng Chang, Yao Cheng, Xin-chun Wang, Jing-ping Zhang, Xiao-juan Feng, Yi-ting Guo

**Affiliations:** Department of Pharmacy, Second Hospital of Shanxi Medical University, Shanxi 030000, China

**Keywords:** GPolycystic ovary syndrome, Hyperandrogenism, Inflammation, nuclear factor kappaB, Shaoyao-Gancao Decoction

This article is being retracted from *Bioscience Reports* at the request of the Editor-in-Chief and the Editorial Board. This follows the receipt of a notification from a reader, alerting the Editorial Board to irregularities in the appearance of the B-actin western blots of Figure 7A, where no background details are visible. The Editorial Office also notes that two further image concerns are present in Figure 5: there is an overlapping region between the Figure 5Cd IL-6 panel and 5Df IL-18 panel, along with overlaps between the Figure 5Bb IL-1B panel and its x400 magnification panel that don't show the expected differences in magnification.

The authors were contacted regarding the concerns raised and provided replacement images for Figure 5; however, images of the original paraffin blocks and slides were not available. They explained that the errors were made due to accidental misplacement of images during figure preparation. Raw data were provided for Figure 7A, and the authors explained that the lack of background detail was a result of the dark-field imaging method used. They also recognised that adjustments made to the brightness and contrast of the B-actin bands specifically were inappropriate.

The raw data provided were assessed by the Editorial Board and concerns remain due to lack of background detail at varying exposure levels as well as lack of visible membrane edges. The Editorial Board therefore stands by the decision to retract. The authors agree to the retraction.

